# Factors that influence midwifery students in Ghana when deciding where to practice: a discrete choice experiment

**DOI:** 10.1186/1472-6920-13-64

**Published:** 2013-05-04

**Authors:** Peter Ageyi-Baffour, Sarah Rominski, Emmanuel Nakua, Mawuli Gyakobo, Jody R Lori

**Affiliations:** 1 School of Medical Sciences, Kwame Nkrumah University of Science and Technology, Kumasi, Ghana; 2 Global REACH, University of Michigan Medical School, Ann Arbor, MI, USA; 3 Medical School, University of Ghana, Accra, Ghana; 4 University of Michigan School of Nursing, Ann Arbor, MI, USA

## Abstract

**Background:**

Mal-distribution of the health workforce with a strong bias for urban living is a major constraint to expanding midwifery services in Ghana. According to the UN Millennium Development Goals (MDG) report, the high risk of dying in pregnancy or childbirth continues in Africa. Maternal death is currently estimated at 350 per 100,000, partially a reflection of the low rates of professional support during birth. Many women in rural areas of Ghana give birth alone or with a non-skilled attendant. Midwives are key healthcare providers in achieving the MDGs, specifically in reducing maternal mortality by three-quarters and reducing by two-thirds the under 5 child mortality rate by 2015.

**Methods:**

This quantitative research study used a computerized structured survey containing a discrete choice experiment (DCE) to quantify the importance of different incentives and policies to encourage service to deprived, rural and remote areas by upper-year midwifery students following graduation. Using a hierarchical Bayes procedure we estimated individual and mean utility parameters for two hundred and ninety eight third year midwifery students from two of the largest midwifery training schools in Ghana.

**Results:**

Midwifery students in our sample identified: 1) study leave after two years of rural service; 2) an advanced work environment with reliable electricity, appropriate technology and a constant drug supply; and 3) superior housing (2 bedroom, 1 bathroom, kitchen, living room, not shared) as the top three motivating factors to accept a rural posting.

**Conclusion:**

Addressing the motivating factors for rural postings among midwifery students who are about to graduate and enter the workforce could significantly contribute to the current mal-distribution of the health workforce.

## Background

A health worker with midwifery skills should be present at every birth according to the joint statement by WHO/UNFPA/UNICEF/World Bank [1]. The UN’s Millennium Development Goal (MDG) 5, set in 2000, targeted a 75% reduction in the maternal mortality ratio by 2015. Great strides have been made toward reaching MDG5 [2]. Despite significant investments in resources and targeted interventions and a 56% reduction of maternal mortality in sub-Saharan Africa between 1990 and 2010, progress towards MDG5 has slowed in recent years and this target will most likely not be met in many countries [3].

Ghana has extreme need in areas related to maternal health. Maternal death is currently estimated at 350 per 100,000, in part a reflection of the low rates of professional or skilled support during childbirth [4]. While there is high uptake of prenatal care, estimated as high as 94 percent, many women in Ghana give birth alone or with a non-skilled attendant [5,6]. Ghana’s health worker density, estimated at 91 per 1,000 population, falls far below the WHO recommended level of 2.28 health care professionals per 1,000 population [7]. Health worker density is negatively associated with maternal mortality and potentially child mortality [8]. If access to skilled a birth attendant improves, women’s lives could be saved and morbidity drastically reduced.

In rural areas of Ghana, poor road conditions, long distances to a health facility, and lack of transportation make accessing health care services a challenge. Contributing to the lack of access, many health workers are unwilling to locate to rural areas where the majority of Ghanaians live [9]. The lack of access to a skilled birth attendant adds to the non-uniform distribution of maternal mortality in Ghana. According to the State of the World’s Midwives (SOWM) report [4], Ghana has reduced maternal mortality by 44% since 1990, but the rate remains high, in large part because of limits to access in care driven by the lack of adequate numbers of midwives.

Identified as a key priority for the Ghana Ministry of Health, the SOWM [4] report highlights the need to recruit and retain midwives especially in the rural northern portion of the country. Differences between urban and rural areas are striking. While less than 20% of urban births were attended by untrained personnel, over 60% of births in rural areas were attended by someone with no formal training. Recognizing this problem, in 2005, the Ministry of Health targeted improving basic obstetrical care training for midlevel providers [10]. As part of this commitment, the Ministry of Health accredited and opened 14 new midwifery training colleges.

Various strategies, including educational interventions such as selecting students with rural background, increasing financial incentives, and professional development incentives, have been implemented in an attempt to recruit and retain health professionals to rural and remote areas [11-13]. These strategies have been poorly evaluated and largely unsuccessful at reversing within-country maldistribution of the health workforce. Understanding the preferences of future midwives for posting to rural, deprived areas is key to achieving the MDGs, specifically in reducing maternal mortality by three-quarters and reducing by two-thirds the under 5 child mortality rate by 2015.

The purpose of this study is to further our understanding of the factors that motivate professional midwifery students to move to rural areas with the final goal of assisting the government in Ghana to develop an incentive package to improve health worker distribution.

## Methods

Used for many years in market research, one tool with potential to suggest working condition priorities is conjoint, or trade-off, analysis. This constellation of techniques provides the researcher with the ability to elicit individuals’ stated preferences. A common form of conjoint analysis is the discrete choice experiment (DCE), in which respondents (e.g. midwifery students) are presented with a choice of several competing hypothetical job posting scenarios each characterized by a variety of attributes (e.g., salary, housing, offer of a car allowance). Respondents are then asked to select their preferred scenario. The benefit of this model is that of opportunity cost. We know individuals want the best of everything, but when resources are limited, the DCE gives a weighted relevance to distinguish which attributes are the most highly incentivizing [14] to motivate individuals to locate to rural areas. Well established in the use of inferring patients’ preferences, this technique has recently been used to test providers’ preferences as well [15].

**Figure 1:**
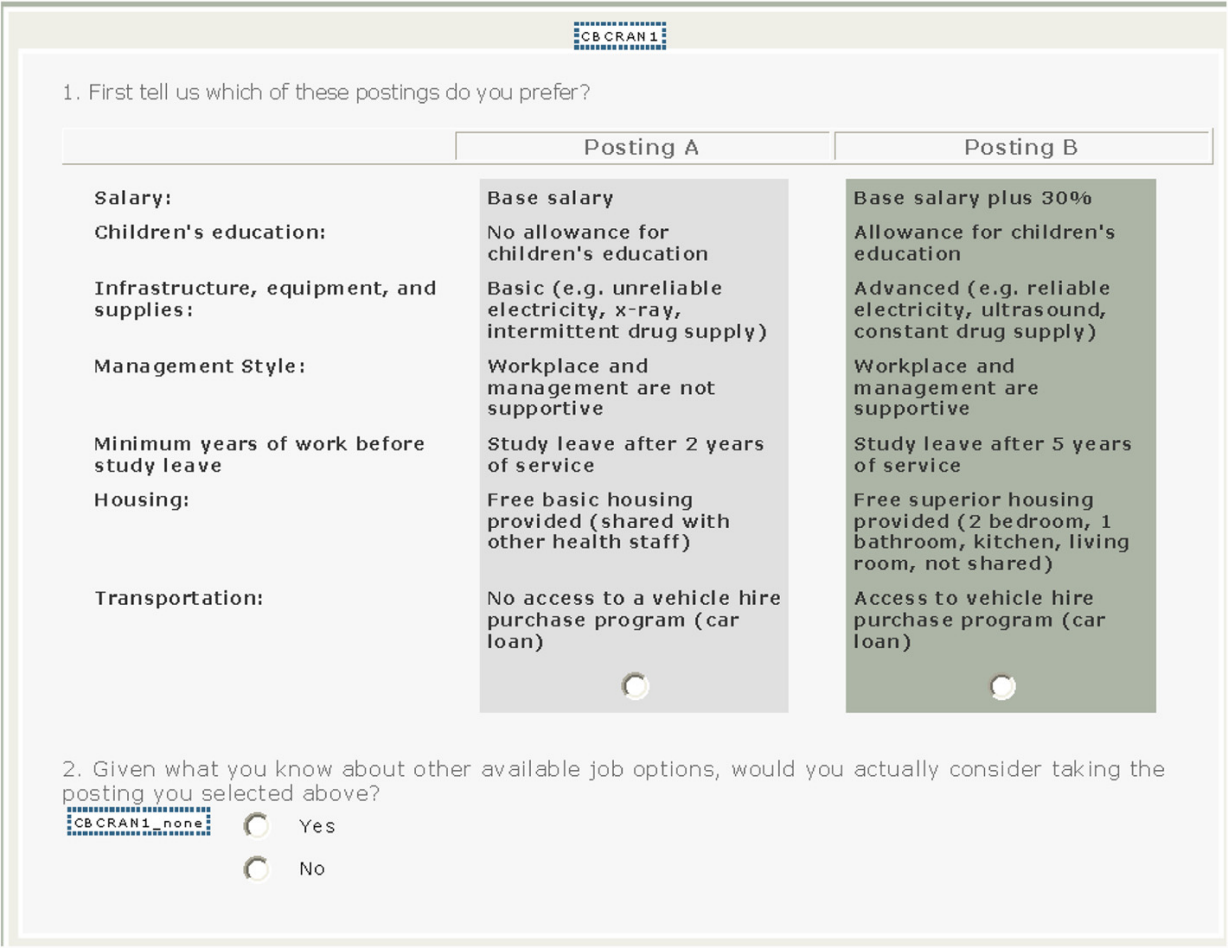
Sample DCE question.

In designing the DCE, we selected attributes (motivators) to increase attraction to rural practice based on conversations with the Ghana Ministry of Health and eight focus groups with final-year midwifery students (n = 49) at the two largest Ghanaian midwifery training colleges. The seven attributes identified by content analysis [16] included: salary, study leave, housing, supportive management, infrastructure, transportation and children’s education. The DCE was then designed to estimate the relative value or utility of different work conditions that might incentivize students to locate to rural areas to practice after graduation. The survey consisted of demographic and background questions followed by a series of 11 discrete choice questions. In these questions, students were asked to compare two hypothetical job postings (Figure 1).

Participants were asked to imagine that upon completion of their midwifery training, they were offered two postings in two rural deprived areas by the Ministry of Health. Deprived area was defined as an area that is distant from a big city with few social amenities such as schools, roads, or pipeborne water. Participants were asked to imagine themselves making a real decision between two rural postings and were asked of the two offered, which they felt was better. Further, students were asked to answer whether or not they would accept this posting if it were offered.

### Setting and sample

Midwifery education in Ghana is a three-year, post-secondary school diploma program. Since 2003, fourteen midwifery training schools in Ghana have been accredited. Of the ten regions in Ghana, each is home to at least one midwifery education program. There is a national curriculum with the first three semesters focused on general nursing and the final three semesters devoted to midwifery knowledge and skills. Students spend at least one clinical rotation at a rural district hospital.

We chose third-year midwifery students (n = 238) about to graduate and considering employment perspectives for our sample. We used purposive sampling to obtain a wide diversity of experiences and opinions. Students at two of the largest midwifery training schools in Ghana were invited to participate in the study. These two schools combined graduate the largest number of midwifery students per year. This survey was part of a larger collaboration between the University of Michigan, the Kwame Nkrumah University of Science and Technology and the Ghana Ministry of Health. The research was approved by the Ghana Health Service Ethical Review Committee, the Kwame Nkrumah University of Science and Technology Committee on Human Research, Publications and Ethics, the University of Ghana Medical School, and the University of Michigan Ethical Review Board.

### Data collection

Informed consent was obtained prior to participation in the DCE. Each computerized survey took approximately 30–45 minutes. Students were given an incentive of 10 Ghana Cedis (approximately 7 US dollars) upon completion of the survey. Students signed in and the names were compared to a class list generated by the head of each college to determine response rate.

### Data analysis

Sawtooth Software (Orem, UT) was used to construct, field and score the surveys. Using market simulator software in Sawtooth’s Choice-Based Conjoint with Hierarchical Bayes module, we used individual-level utilities to estimate the proportion of respondents who would prefer specific incentive packages. The software calculates total utilities of the simulated options for each respondent by summing attribute utilities. The respondents were repeatedly sampled to stabilize these preferences. In addition, we added a random error term to the estimates of utilities to correct for any similarities in scenarios. We used Sawtooth’s Choice-Based Conjoint with Hierarchical Bayes statistical program to estimate coefficients for the individual utilities of each attribute level.

## Results

Two-hundred and thirty eight upper level midwifery students completed our survey for a response rate of 79.8%. See Table 1 for select demographic data.

**Table 1:** Select demographic data

**Characteristics (n = 238)**	**Mean or No (%)**
Age	24.5 years
	Range (18–33 years)
Gender	
Female	238 (100%)
Male	0 (0%)
Marital Status	
Married	8 (3.4%)
Living with a partner	4 (1.7%)
In a relationship, not living together	135 (56.7%)
Not in a relationship	89 (37.4%)
Rather not say	2 (0.8%)
Number of Children	
None	227 (95.4%)
One	1 (0.4%)
Two	1 (0.4%)
Three	2 (0.8%)
Ever lived in a rural area	
Yes	91 (38.2%)
No	146 (61.3%)
Don’t know	1 (0.4%)
Birth Location	
Urban	168 (70.6%)
Periurban	42 (17.6%)
Rural	19 (8%)
Don’t know	7 (2.9%)
Rather not say	.2 (0.8%)

While less than 5% of our respondents currently had children, nearly all (99.2%) reported they plan to have children in the future. The vast majority of the sample (98.7%) attended public high school with only 1.3% (n = 3) reporting they attended private school prior to midwifery training. Almost half (47.1%) would like to be practicing general midwifery in 10 years with the remainder reporting they preferred a position in administration (21.8%), public health (13.9%), teaching (7.1%) or nursing (1.7%). One hundred and seventy seven (74.4%) respondents reported they believe midwives are “very valued” in their society, with another 23.5% reporting that midwives are “somewhat valued”. Only 2.1% reported that midwives are either somewhat or very unvalued. The vast majority of our sample (99.3%) reported they would like to return to university in the future to pursue a university degree.

### Discrete choice experiment

The full results of the DCE can be seen in Table 2. For each of the parameter estimates, the currently offered level was used as the reference.

**Table 2:** DCE results

	**Parameter**		**95%**	**CI**
**Salary**^**1**^	Mean	0.72	0.60	0.84
	SD	0.99		
**Allowance for Children's education**^**2**^	Mean	−0.93	−1.03	−0.83
	SD	0.83		
**Basic Infrastructure, equipment, and supplies**^**3**^	Mean	−1.07	−1.20	−0.93
	SD	1.11		
**Management are supportive**^**4**^	Mean	0.96	0.85	1.07
	SD	0.93		
**2 years before study leave**^**5**^	Mean	1.24	1.02	1.45
	SD	1.78		
**No Housing**^**6**^	Mean	−1.88	−2.05	−1.71
	SD	1.39		
**Superior Housing**^**6**^	Mean	−0.87	−0.99	−0.75
	SD	1.02		
**No access to car**^**7**^	Mean	−0.88	−0.98	−0.77
	SD	0.89		

1 continuous variable; coefficient represents the magnitude of increase in utility for every 10% increase in salary.

2 compared to no allowance for children’s education.

3 compared to improved infrastructure, equipment, and supplies.

4 compared to unsupportive workplace and management.

5 compared to five years minimum work before study leave.

6 compared to basic housing.

7 compared to access to a utility car.

In our sample, the highest parameter estimates for rural placement by third year midwifery students were study leave after two years versus after five years, and having an “advanced” work environment (reliable electricity, ultrasound, constant drug supply) with utilities of 1.24 and 1.07. Superior housing, defined as a two bedroom, one bath, not shared house) also had a relatively high parameter estimate of 0.87. In contrast, providing no housing to midwifery students in rural area negatively influenced their decision to practice in underserved areas.

Increasing salary had a utility of 0.72 and access to a vehicle hire program had a utility of 0.88. Having no incentives structure in place had a large negative utility at −3.67.

### Limitations

As only two schools, both located in major urban areas, were sampled, it is not clear to what extent the findings can be extrapolated to all midwifery students in Ghana. However, it is plausible that those students studying in Accra and Kumasi would be more reluctant to locate to rural areas, as they are accustomed to living and studying in well-appointed areas.

## Discussion

There is a great need to improve the provision of maternal and child health in rural Ghana. One important cadre of worker that can deliver this necessary care is midwives. Finding incentives to motivate these midwives to locate to rural areas is of the utmost importance.

We explored the factors and their strengths that influence reported acceptance to rural postings among graduating midwives. The top three factors identified include: 1) study leave after two years versus five years of working in rural areas; 2) advanced working conditions such as electricity, regular drug supplies, and equipment, and; 3) a free superior housing scheme. These results corroborate those found by Kruk and colleagues [15] among Ghanaian medical students and Kwansah and colleagues [17] among Ghanaian nursing students.

Considering Ghana has recently expanded midwifery training by starting the first bachelor’s degree in midwifery, educational incentives offer a promising direction for future interventions. However, study leave after two years of service will ultimately impact the training schools. The needs of these schools must to be taken into account when thinking through potential interventions, as many schools in Ghana are currently at or past capacity.

Providing working conditions that offer a full range of amenities to health workers is a priority of the government of Ghana. In qualitative work with a subset of midwifery students, the need for professional, as well as personal, support was noted [16]. This level of on-going support requires intense planning and deployment efforts by governmental agencies to realize.

The strong desire identified by our sample for free superior housing and the negative utility of no housing suggests these students are aware of poor housing options in rural areas and consider free superior housing a requirement for accepting a rural posting. This finding is consistent with previous work in Ghana and Ethiopia which shows other cadres of students are also motivated by the availability of housing [15,18-20]. Providing a free housing scheme requires cooperation between the health ministry, local government, and economic planning ministries. Thus the need for intersectoral collaborations in the recruitment and retention of health staff in rural areas becomes eminent.

Interestingly, increased salary was not as important to these students as expected. Increased salary lagged behind educational, professional and housing interventions in our sample. Work in Ethiopia has suggested that for physicians, large salary increases are needed to motivate work in deprived areas [18]. It does not appear from these results that increasing salary will be as motivating for young midwives as improving the clinic amenities, offering superior housing, or enabling a return to school.

Allowances for children’s education were among the least motivating incentives in the policy packages. Considering the majority of participants in this study planned on having children in the future, this is somewhat surprising. Perhaps, as Kruk and colleagues [15] found, given the young age of the participants and their plans to have children in the future, the well-being of these future children does not factor as heavily as the participants’ own well-being.

This research points to future research including a policy experiment testing the packages that were most attractive to these participants. The enormous shortage of health care workers practicing in rural Ghana, and other low-income countries, points to the importance of carrying out these kinds of policy experiments. The simulation presented in this paper can offer a starting point to create a series of packages for experimentation.

## Conclusion

Maternal death remains high and one of the top public health concerns in Ghana. With barely three years to go for the UN MDGs, there is a huge need for uncompromising effort to improve access to care in rural areas. If the global strides made in reduction of maternal deaths are to be sustained [21] and the targeted 75% reduction in the maternal mortality ratio by 2015 achieved [1], greater access to skilled care in rural Ghana is needed.

Findings from our study conclude the three most important factors graduating midwives consider in accepting postings to rural underserved areas include study leave after two years versus five years of working in rural areas, advanced working condition and free superior housing scheme. Without addressing these needs to correct the mal-distribution of skilled health staff in urban and rural areas, Ghana is unlikely to realize the goals of MDG 5.

## Competing interests

The authors declare that they have no competing interests.

## Authors’ contributions

PAB was involved in all aspects of the study including conceptualization of the study, development of the study instruments, data collection, data analysis, drafting and revising of the manuscript. SR was involved in all aspects of the study including conceptualization of the study, development of the study instruments, data collection, data analysis, drafting and revising of the manuscript. EN was involved in data collection, data analysis and drafting and revising of the manuscript. MG was involved in data collection and drafting and revising of the manuscript. JL was involved in all aspects of the study including conceptualization of the study, development of the study instruments, data collection, data analysis, drafting and revising of the manuscript. All authors read and approved the final manuscript.

## Pre-publication history

The pre-publication history for this paper can be accessed here:

http://www.biomedcentral.com/1472-6920/13/64/prepub
